# A TCN-Linear Hybrid Model for Chaotic Time Series Forecasting

**DOI:** 10.3390/e26060467

**Published:** 2024-05-29

**Authors:** Mengjiao Wang, Fengtai Qin

**Affiliations:** School of Automation and Electronic Information, Xiangtan University, Xiangtan 411105, China; 202121622930@smail.xtu.edu.cn

**Keywords:** chaos prediction, time series forecasting, neural networks

## Abstract

The applications of deep learning and artificial intelligence have permeated daily life, with time series prediction emerging as a focal area of research due to its significance in data analysis. The evolution of deep learning methods for time series prediction has progressed from the Convolutional Neural Network (CNN) and the Recurrent Neural Network (RNN) to the recently popularized Transformer network. However, each of these methods has encountered specific issues. Recent studies have questioned the effectiveness of the self-attention mechanism in Transformers for time series prediction, prompting a reevaluation of approaches to LTSF (Long Time Series Forecasting) problems. To circumvent the limitations present in current models, this paper introduces a novel hybrid network, Temporal Convolutional Network-Linear (TCN-Linear), which leverages the temporal prediction capabilities of the Temporal Convolutional Network (TCN) to enhance the capacity of LSTF-Linear. Time series from three classical chaotic systems (Lorenz, Mackey–Glass, and Rossler) and real-world stock data serve as experimental datasets. Numerical simulation results indicate that, compared to classical networks and novel hybrid models, our model achieves the lowest RMSE, MAE, and MSE with the fewest training parameters, and its R^2^ value is the closest to 1.

## 1. Introduction

Chaotic research constitutes an interdisciplinary field [1], encompassing theories of dynamical systems, nonlinear dynamics, and complex systems [2]. The significance of chaos theory lies in its elucidation of non-periodic behaviors and unpredictable characteristics within numerous systems [3]. Notably, chaotic systems demonstrate extreme sensitivity to initial conditions, where minor initial discrepancies can lead to significant deviations in system trajectories, resulting in long-term unpredictability while retaining short-term predictability [4]. This phenomenon marks chaos as a key feature of complex system behaviors, offering new perspectives for our understanding and analysis of these systems [5].

Time series forecasting involves predicting future outputs using historical information and future input signals [6], reflecting the dynamic changes of the system in the future, and holds broad research prospects [7]. In the classical forecasting domain, mathematical models with rigorous derivations offer good interpretability; however, unknown parameters in the system increase the difficulty of modeling [8]. Linear or single-degree-of-freedom dynamic systems are easier to predict, whereas, due to their sensitivity to initial conditions—characteristic of chaos—chaotic time series are more challenging to forecast [9].

The advent of data-driven modeling techniques has posed challenges for researchers, while the evolution of neural networks, particularly deep learning, bolstered by advancements in computer hardware, has opened new avenues for automated data analysis. Amaranto et al. [10], for instance, developed B-AMA (Basic dAta-driven Models for All), a flexible and easy-to-use tool for both non-expert users and more experienced developers. As for deep learning, traditional Convolutional Neural Networks (CNNs) [11] have been proven effective in the domain of image recognition, while Recurrent Neural Networks (RNNs), due to their unique neural unit structure, exhibit superior performance in processing sequential data [12]. Despite many RNN variants optimizing their performance by introducing gating mechanisms, issues such as vanishing or exploding gradients and high computational costs due to the inability to compute in parallel still persist [13]. Consequently, since the introduction of the Transformer [14] network in 2017, its parallel processing capabilities based on the attention mechanism and its ability to capture long-distance dependencies have demonstrated remarkable abilities in natural language processing tasks, making it and its variants the focus of current neural network research. However, Zeng et al. questioned the Transformer network’s capabilities in time series forecasting [15]. They pointed out that the attention mechanism could lead to the loss of temporal scale information, which is crucial for time series forecasting. They introduced a structurally simple LSTF-Linear network that outperformed the Transformer network on multiple test sets.

The study of time series forecasting for dynamic system states holds practical value [16]. In recent decades, a considerable amount of research has been dedicated to applying neural networks to chaotic time series forecasting and related application domains. Sun et al. [17] trained an encoder–decoder with LSTM units to perform chaos forecasting on a five-degree-of-freedom duffing oscillator system. The numerical simulations revealed that the LSTM ED model can accurately predict chaotic time series with limited data, achieving a prediction window twice the size of the observation window. Uribarri et al. [18] found out that under certain conditions, Long Short Term Memory networks can learn to forecast time series from chaotic systems by generating an embedding in their inner state that is topologically equivalent to the original strange attractor. Sangiorgio et al. [19] implemented a multi-step approach to predict an entire interval of future values, and compared the performances of various neural network architectures in real-world cases. Ref. [20] recommends training LSTM networks without the teacher forcing them to improve accuracy and robustness, ensuring a more uniform distribution of the predictive power within the chaotic attractor. Ref. [21] found that recurrent architectures of networks are more suitable for learning the non-stationary dynamics caused by structural noise. Pathak et al. [22] presented a parallel scheme with an example implementation based on the Reservoir Computing (RC) paradigm that offered a new direction in the field of chaos prediction. Ref. [23] found that RNNs trained via backpropagation through time show superior forecasting abilities and capture the dynamics of reduced order systems well. Patel et al. [24] found that machine learning shows excellent performance in predicting the long-term behavior of a non-stationary dynamical system.

In recent studies, hybrid models that leverage the strengths of various models through parallel or sequential combinations have demonstrated superior performance, which is emerging as a new trend in research. Xia et al. [25] presented a stacked GRU-RNN for the prediction of renewable energy generation and electricity load; the experimental results demonstrated that the models achieved an accurate energy prediction for effective smart grid operation. Lazcano et al. [26] combined the characteristics of a GCN and a Bi-LSTM network to forecast the price of oil and reached a low error of RMSE, MSE, and MAPE. Cao et al. [27] established a new hybrid time series forecasting method by combining the EMD and CEEMDAN algorithms with the LSTM neural network for financial time series, which exhibited a more accurate predictive performance than other similar models. Fu et al. [28] designed a deep temporal inception module and gated recurrent unit network (DTIGNet), which demonstrated superior accuracy and performance in predicting chaotic time series, outperforming other methods based on six evaluation metrics. The significance of chaotic time series prediction also extends to its application in fields such as weather forecasting [29,30], the stock market [31,32], wind power [33,34], and traffic flow analysis [35,36], highlighting its critical role across various domains.

Building on the analysis provided, we introduce a novel hybrid neural network architecture, the TCN-Linear model. This model harnesses the advantages of dilated and causal convolutions within the TCN network, thereby expanding the model capacity of LSTF-Linear and enhancing its specialized learning capabilities for diverse time series data. It circumvents the gradient issues associated with RNNs and the loss of temporal scale information attributed to the attention mechanisms in Transformers, achieving a higher prediction accuracy with a reduced parameter count for training. The remainder of the paper is organized as follows. Section 2 introduces the theoretical underpinnings and the development of the model. Section 3 describes the experimental setup and results. Section 4 concludes the work and offers perspectives on future research.

## 2. Proposed Model

### 2.1. TCN

Bai et al. [37] introduced a novel Temporal Convolutional Network (TCN) that adapts convolutional networks for the processing of time series data. This model leverages a proposed causal convolution method to capture local dependencies within sequence data, ensuring temporality, and employs dilated convolutions to expand its receptive field for better learning of data correlations. Additionally, it utilizes convolutional operations for efficient parallel computation, making it suitable for large-scale data processing.

#### 2.1.1. Causal Convolution

**Figure 1:**
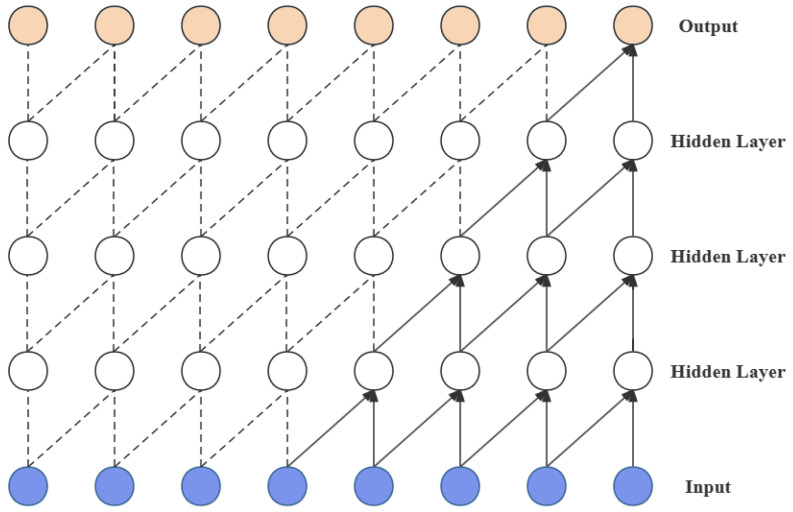
Structure of causal convolution.

Causal convolution, as shown in Figure 1, is one of the core concepts of TCN. To ensure that convolution operations only utilize past information, causal convolution employs zero-padding at the beginning of the sequence. This technique ensures that the output at each time step is influenced solely by that point and its preceding inputs. Such an approach prevents forward leakage of information and maintains temporal alignment between the input and output sequences.

#### 2.1.2. Dilated Convolution

**Figure 2:**
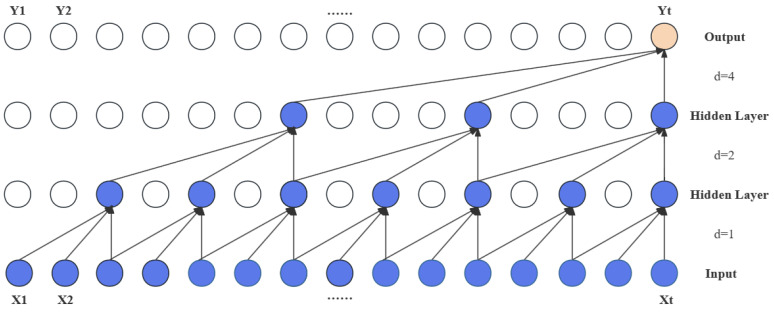
Structure of dilated convolution.

Dilated convolution represents another crucial component within TCN, serving as an extension of traditional convolution aimed at enlarging the receptive field of the convolutional layers. This enlargement enables the network to capture temporal dependencies over longer ranges. In dilated convolution, zeros are inserted between the elements of the convolution kernel (i.e., the dilation rate), allowing the network to cover a larger input area without an increase in the number of parameters or the computational complexity; the structure of dilated convolution is shown in Figure 2.

With the increase in network depth, the dilation rate can be progressively increased, enabling deeper convolutional layers to possess an extensive receptive field. Consequently, the network can effectively learn long-term temporal dependencies without sacrificing temporal resolution.

#### 2.1.3. Residual Connection

**Figure 3:**
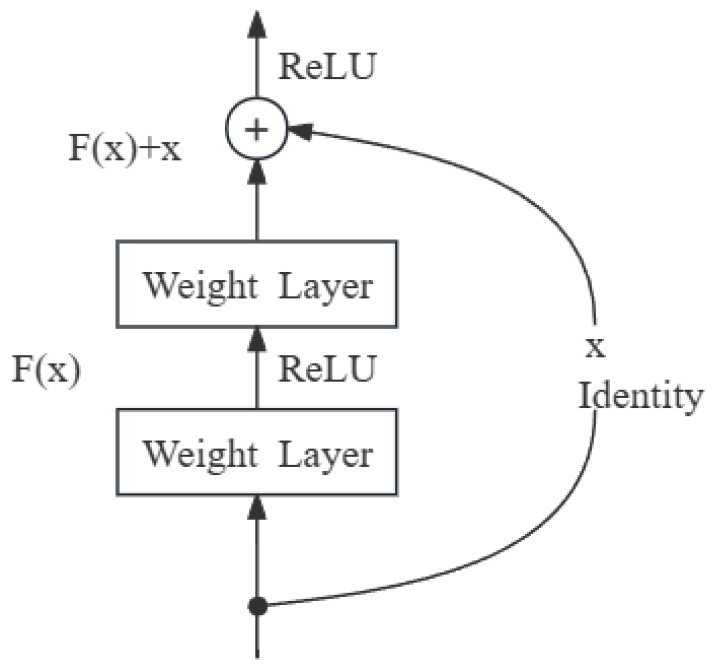
Structure of residual connection.

TCN mitigates the effects of gradient vanishing and explosion in deep networks to some extent. This model introduces straightforward direct connection channels, allowing the network to learn identity mapping, as shown in Figure 3. This ensures that the performance of deep networks does not degrade more than that of their shallower counterparts, and prevents the initial data weight increase caused by dimension changes during the input processing phase.

### 2.2. LSTF-Linear

**Figure 4:**
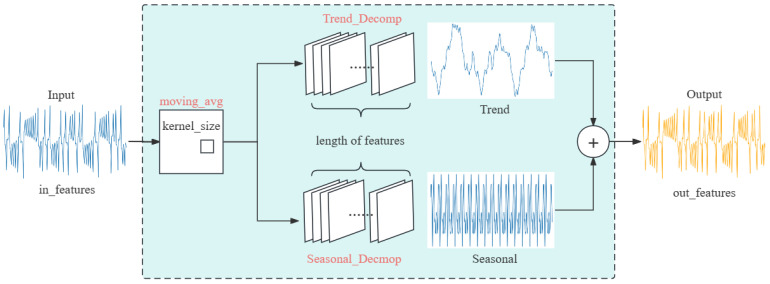
Structure of D-Linear.

LSTF-Linear is a simple direct multi-step model that operates via a temporal linear layer. The fundamental approach of LTSF-Linear employs a weighted sum operation to directly predict future values by regressing on historical time series data (as illustrated in Figure 4). The mathematical expression is $\hat{X_{i}}=WX_{i}$, where $W\in R^{T\times L}$ is a linear layer along the temporal axis. $\hat{X_{i}}$ and $X_{i}$ are the prediction and the input for each i variate.

Specially, D-Linear (shown in Figure 4) is a hybrid of a seasonal decomposition encoder–decoder and the Linear network which decomposes the original data into seasonal and trend components through a moving average kernel. Subsequently, each component is processed using a single linear layer, and the output results are summed to obtain the final prediction. This strategy enhances the model’s performance when the data exhibit clear trends, which coincidentally can be identified within the phase diagrams of chaotic systems.

### 2.3. TCN-Linear

**Figure 5:**
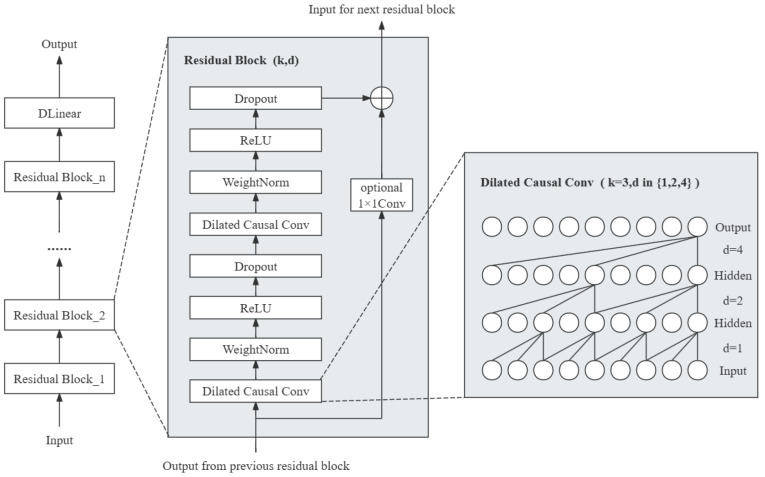
Structure of TCN-Linear.

To further improve model capacity and prediction accuracy, a new hybrid model for chaotic time series prediction is proposed in this paper, named TCN-Linear, which is shown in Figure 5. We tried to improve the structure of D-Linear by fusing it with TCN. This model is constructed with several Residual Block modules and a D-Linear network, where each Residual Block contains two Dilated Causal Conv, two WeightNorm layers, two ReLu layers, and two Dropout layers. In the Dilated Causal Convolution within these blocks, the dilation factor (d) is set to values in the set {1,2,4} and the output of each current layer will serve as the input of the next layer. Finally, the prediction is output by the combination of the Decomposition scheme and the Linear layers.

This hybrid architecture is designed to address the complexities of time series data that exhibit both long-term dependencies and seasonal patterns, making the model versatile across different time series forecasting tasks. Furthermore, TCNs offer efficient parallel computation, significantly reducing both the number of training parameters and the training time compared to traditional RNN- and Transformer-based solutions. This efficiency, when combined with the direct computational characteristics of LSTF-Linear networks, renders the TCN-Linear model particularly suitable for large-scale time series datasets.

In the model, we employed the mean squared error (MSE) as the loss function—a commonly used metric in regression problems that calculates the mean squared error between the predicted values and the actual values. The Adaptive Moment Estimation (ADAM) optimizer [38] was utilized for network training due to its efficiency, robustness, and ease of configuration, making it one of the preferred optimizers in deep learning applications. Its role is to adjust the network parameters to minimize the loss function. Additionally, we adopted early stopping to prevent overfitting. This technique terminates the training process prematurely when the validation error stops decreasing after a certain number of epochs, thereby ensuring the model’s generalization capability.

## 3. Experimental Evaluation

In this section, we evaluate the predictive capability, training cost, and applicability to real financial data of the proposed model using three classical chaotic systems (the Lorenz system, the Mackey–Glass system, and the Rossler system) and real-life stock data.

### 3.1. Dataset

#### 3.1.1. Lorenz

The Lorenz equations were introduced in 1963 by Edward N. Lorenz [39] during his research on atmospheric convection, marking the inception of chaotic research. The Lorenz model is a dynamic system comprising three ordinary differential equations, representing the three-dimensional state of convective rolls.

(1) $$\left\{\begin{array}{l} \frac{dx}{dt}=-\sigma (x-y)\\ \frac{dy}{dt}=-xz+rx-y\\ \frac{dz}{dt}=xy-bz \end{array}\right.$$

when the parameters are set to *σ* = 10, *b* = 8/3, *r* = 28, the system behaves in a chaotic state. In this state, with initial values set to *x*(0) = 1, *y*(0) = 0, and *z*(0) = 1, we generated time series for the system’s three variables by employing the ODE45 integration method at a sampling frequency of 200 Hz within the time interval (*t* in [0, 55]), which had 11,000 points. We removed the first 3000 transient values and divided the remaining 8000 data points into training, validation, and test sets in a 6:2:2 ratio.

#### 3.1.2. Mackey–Glass

The Mackey–Glass system, introduced in 1977 by Michael C. Mackey and Leon Glass [40], is a delay differential equation frequently utilized as a benchmark in chaotic time series analysis.

(2) $$\dot{x}\left(t\right)=-bx\left(t\right)+\frac{ax\left(t-\tau \right)}{1+x^{c}\left(t-\tau \right)}$$

when the parameters are set to *a* = 0.2, *b* = 0.1, *c* = 10, *τ* = 17, the system behaves in a chaotic state. In this state, we generated a time series for the system by employing the ODE45 integration method at a sampling frequency of 10 Hz within the time interval (*t* in [0, 1100]), which had 11,000 points. We removed the first 3000 transient values and divided the remaining 8000 data points into training, validation, and test sets in a 6:2:2 ratio.

#### 3.1.3. Rossler

The Rossler model, introduced in 1976 by the German biophysicist Otto E. Rössler [41], is a chaotic system. Compared to the Lorenz model, the equations of the Rossler model are simpler, and its phase diagram exhibits a clear spiral structure. This demonstrates that complex chaotic behavior can be observed even in exceedingly simple systems.

(3) $$\left\{\begin{array}{l} \frac{dx}{dt}=-(y+z)\\ \frac{dy}{dt}=-x+ay\\ \frac{dz}{dt}=b+z(x-c) \end{array}\right.$$

when the parameters are set to *a* = 0.2, *b* = 0.2, *c* = 5.9, the system behaves in a chaotic state. In this state, with the initial values set to *x*(0) = 0, *y*(0) = 0, and *z*(0) = 0, we generated time series for the system’s three variables by employing the ODE45 integration method at a sampling frequency of 50 Hz within the time interval (*t* in [0, 220]), which had 11,000 points. We removed the first 3000 transient values and divided the remaining 8000 data points into training, validation, and test sets in a 6:2:2 ratio.

#### 3.1.4. Google Stock Price

**Figure 6:**
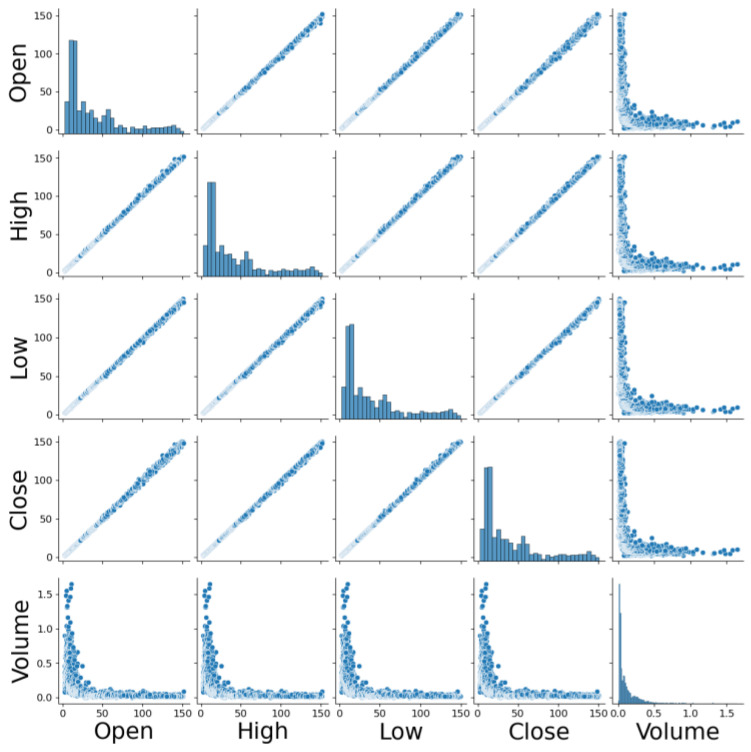
Mutual variables relationship diagram.

We collected stock trading data of Google Inc. (San Francisco, CA, USA) from 2014 to 2024 from public databases. This dataset encompasses key financial indicators over a decade, including the opening price, closing price, highest price, lowest price of the day, and trading volume of Google’s stock. As illustrated in Figure 6, we utilized the pairplot method from the seaborn library to create a diagonal chart that showcases the relationships between multiple variables, and selected the variables strongly correlated with the closing price as features. Subsequently, we divided the 4858 data points into training, validation, and test sets in a 6:2:2 ratio.

### 3.2. Experiment Settings

#### 3.2.1. Experimental Configuration

The main hardware environments of the experiments were as follows: AMD R5-5600X CPU, NVIDIA RTX 3080Ti 16 GB GPU, and 16 GB of RAM, and the operating system of the computer was Windows 10. The software configuration used for simulation experiments in this study included CUDA Version 12.3, GPU Driver Version 546.29, torch 1.9.0+cu111, and python 3.9.18, and the parameter settings are shown in Table 1.

#### 3.2.2. Prediction Evaluation Index

To evaluate the performance of each model, we set the MAE, MSE, RMSE, MAPE, and R^2^, and their definitions are as follows:

(4) $$MAE=\frac{1}{n}\sum_{i=1}^{n}\left|\left(y_{i}-{\hat{y}}_{i}\right)\right|$$

(5) $$MSE=\frac{1}{n}\sum_{i=1}^{n}(y_{i}-{\hat{y}}_{i}{)}^{2}$$

(6) $$RMSE=\sqrt{\frac{1}{n}\sum_{i=1}^{n}(y_{i}-{\hat{y}}_{i}{)}^{2}}$$

(7) $$MAPE=\frac{1}{n}\sum_{i=1}^{n}\left|\frac{y_{i}-{\hat{y}}_{i}}{y_{i}}\right|\times 100$$

(8) $$R^{2}=1-\frac{\sum_{i=1}^{n}(y_{i}-{\hat{y}}_{i}{)}^{2}}{\sum_{i=1}^{n}(y_{i}-\bar{y}{)}^{2}}$$

where *n* is the length of the predicted series, $y_{i}$ is the true value, ${\hat{y}}_{i}$ is the predicted value, and $\bar{y}$ represents the mean of the true value of the sequence, respectively.

### 3.3. Results

#### 3.3.1. Lorenz

**Figure 7:**
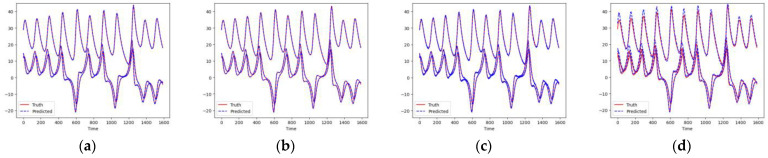
Predicted time series of Lorenz. (**a**) RC; (**b**) TCN-Linear; (**c**) LSTM; (**d**) Transformer.

**Figure 8:**
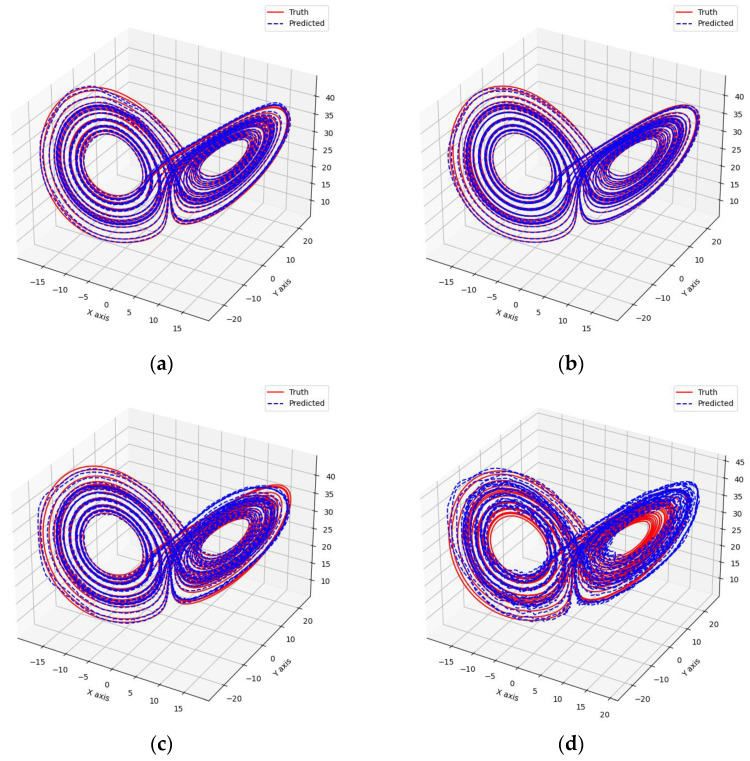
Predicted phase diagrams of Lorenz. (**a**) RC; (**b**) TCN-Linear; (**c**) LSTM; (**d**) Transformer.

**Figure 9:**
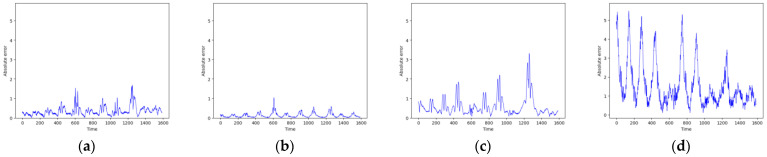
Predicted absolute errors of Lorenz. (**a**) RC; (**b**) TCN-Linear; (**c**) LSTM; (**d**) Transformer.

As shown in Figure 7 and Figure 8, these models can capture the dynamic changes of the Lorenz system effectively. Their blue prediction curves align well with the red actual value curves, except for the Transformer network, which exhibits significant noise and fluctuation in its predictions. The results presented in Figure 9 and Table 2 further suggest that the TCN-Linear network, with the smallest number of training parameters, outperforms the other models to varying degrees across various indexes.

#### 3.3.2. Mackey–Glass

**Figure 10:**
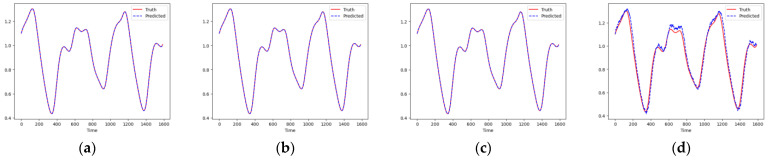
Predicted time series of Mackey–Glass. (**a**) RC; (**b**) TCN-Linear; (**c**) LSTM; (**d**) Transformer.

**Figure 11:**
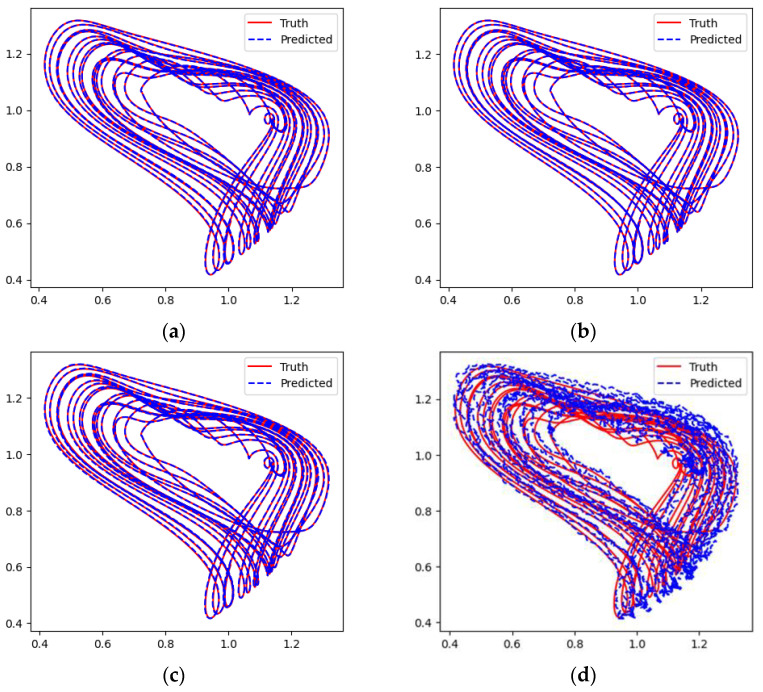
Predicted phase diagrams of Mackey–Glass. (**a**) RC; (**b**) TCN-Linear; (**c**) LSTM; (**d**) Transformer.

**Figure 12:**
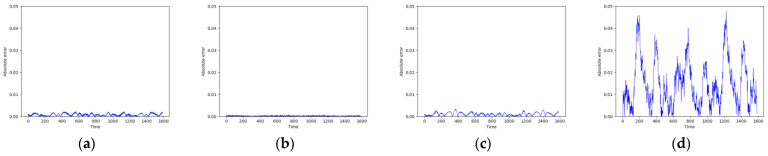
Predicted absolute errors of Mackey–Glass. (**a**) RC; (**b**) TCN-Linear; (**c**) LSTM; (**d**) Transformer.

Due to the Mackey–Glass system’s time series containing only one dimension, the networks can fit its curves more smoothly, as demonstrated in Figure 10. However, in the phase space reconstruction shown in Figure 11, the Transformer network still exhibits more noise compared to the other models. According to the results in Figure 12 and Table 3, the error values of all models are quite low, with TCN-Linear continuing to exhibit the best performance among them.

#### 3.3.3. Rossler

**Figure 13:**
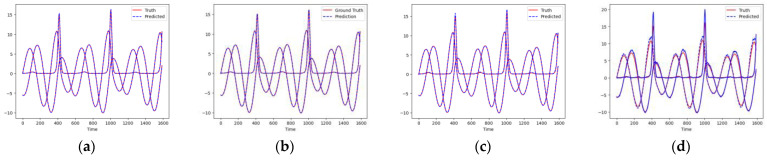
Predicted time series of Rossler. (**a**) RC; (**b**) TCN-Linear; (**c**) LSTM; (**d**) Transformer.

**Figure 14:**
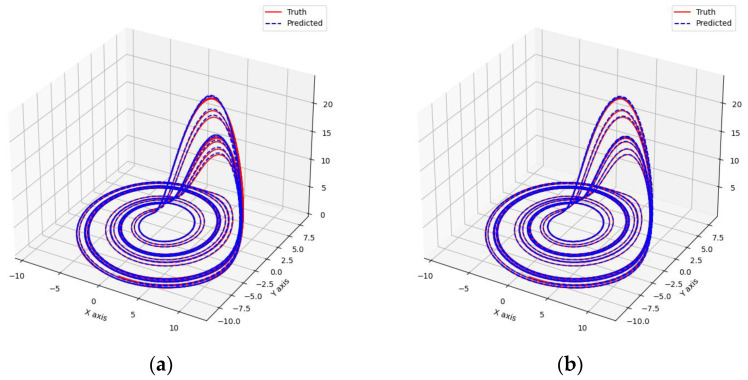

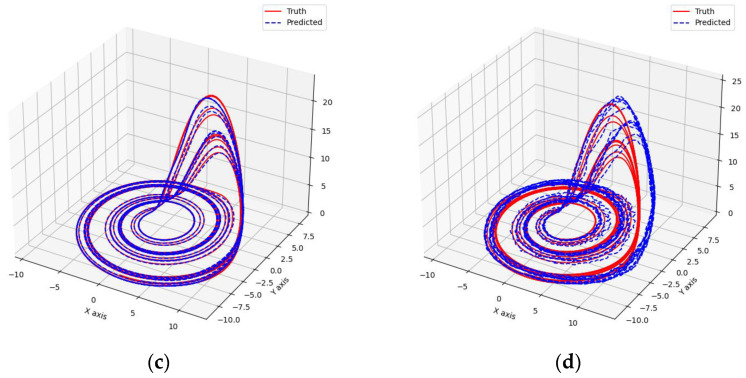
Predicted phase diagrams of Rossler. (**a**) RC; (**b**) TCN-Linear; (**c**) LSTM; (**d**) Transformer.

**Figure 15:**
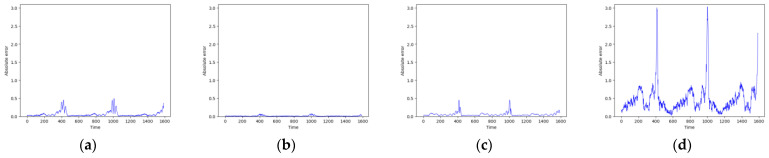
Predicted absolute errors of Rossler. (**a**) RC; (**b**) TCN-Linear; (**c**) LSTM; (**d**) Transformer.

Similar to the previous two experiments, the networks demonstrated excellent performance in fitting the time series of the Rossler system, as can be seen from Figure 13, Figure 14 and Figure 15. In Table 4 the RMSE, MAE, and MSE values were all very low, and the R^2^ values were very close to 1. Once again, TCN-Linear emerged as the top performer.

#### 3.3.4. Google Stock Price

To demonstrate the model’s applicability to real financial sequences, we selected Google’s stock data from the past decade for analysis and compared TCN-Linear’s performance with that of hybrid models CNN-GRU [42], Seq2Seq [43], and Bi-LSTM [44], which have shown promising results in this domain.

**Figure 16:**
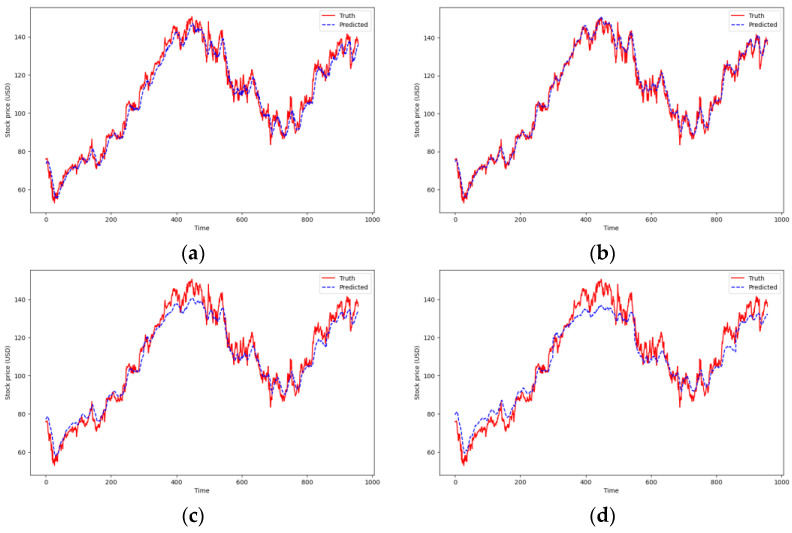
Predicted stock price of Google. (**a**) Bi-LSTM; (**b**) TCN-Linear; (**c**) Seq2Seq; (**d**) CNN-GRU.

Due to the high frequency of fluctuations in real stock data, it is challenging for models to fully learn and fit the actual values without overfitting the data. As depicted in Figure 16, the prediction curves roughly outline the general trend of the stock price. The indexes in Table 5 show a noticeable deterioration compared to the previous chaotic systems; however, the TCN-Linear model still demonstrates excellent predictive capabilities.

## 4. Conclusions

In this paper, a novel hybrid TCN-Linear model for the prediction of chaotic time series is proposed. To enhance the capacity of the LSTF-Linear model, we integrated it with the Temporal Convolutional Network model, which has long-term memory and parallel computing capabilities, thereby circumventing the gradient issues associated with RNNs and the loss of temporal scale information due to the attention mechanisms in Transformers. Experiments conducted on time series generated by several classical chaotic systems and real stock sequences demonstrate that our model is capable of capturing the future trends of dynamic systems and making accurate predictions. It achieved the lowest error metrics compared to other models, with the R^2^ value closest to 1. The novel structure of our model offers fresh insights into solving LTSF problems. However, there are still some limitations in our work. For instance, it is challenging to maintain low error rates in multi-step predictions of multi-dimensional or high-frequency variable data. Moreover, there is still a long way to go in terms of accurately restoring the dynamic behaviors and patterns of chaotic systems. We believe that Recurrent Neural Networks and Reservoir Computing hold promising potential in nonlinear dynamics analysis, so these are areas we aim to explore in future improvements. Our future research will focus on designing models that balance computational resources and prediction accuracy, and applying them to more complex real-world engineering applications such as weather systems, turbulent flow data, and industrial fault diagnosis.

## Figures and Tables

**Table 1:** Hyperparameter settings.

Parameters	Value
seed	42
batch_size	32
in_seq_len	24
out_seq_len	12
num_epochs	500
learning_rate	0.001
es_patience	15
lr_patience	5
kernel_size	25
num_layers	1
hidden_size	64
train_ratio	0.6
teaching_forcing_prob	0.75
dropout	0.2
criterion	nn.MSELoss
optimizer	optim.Adam

**Table 2:** Evaluation scores of Lorenz.

Index	TCN-Linear	Transformer	LSTM	RC
RMSE	0.02595169	1.38334787	0.29731486	0.10683146
MAE	0.01839166	1.03468537	0.19289005	0.07635882
MSE	0.00067349	1.91365147	0.08839613	0.06396773
R^2^	0.99999064	0.97296977	0.99877666	0.99997096
parameter	2853	234,435	17,859	13,689
epoch	240	46	112	101

**Table 3:** Evaluation scores of Mackey–Glass.

Index	TCN-Linear	Transformer	LSTM	RC
RMSE	0.00018653	0.03591761	0.00122464	0.00094763
MAE	0.00014705	0.02931397	0.00101639	0.00083345
MSE	0.00000003	0.00129008	0.00000150	0.00000105
R^2^	0.99999941	0.97798753	0.99997440	0.99996435
parameter	951	234,049	17,217	8735
epoch	76	13	163	69

**Table 4:** Evaluation scores of Rossler.

Index	TCN-Linear	Transformer	LSTM	RC
RMSE	0.01509988	0.52885771	0.06465438	0.04373308
MAE	0.00645125	0.31585521	0.03655762	0.04769310
MSE	0.00022801	0.27969050	0.00418019	0.00374805
R^2^	0.99996984	0.97996159	0.99948332	0.99984731
parameter	2853	234,435	17,859	10,176
epoch	93	27	108	147

**Table 5:** Evaluation scores of the Google stock price.

Index	TCN-Linear	CNN-GRU	Seq2Seq	Bi-LSTM
RMSE	3.820	7.703	6.408	4.366
MAE	2.918	5.730	4.621	4.200
MSE	14.591	16.832	15.973	14.386
R^2^	0.976	0.921	0.964	0.953
MAPE	2.793%	5.964%	4.389%	3.763%

## Data Availability

The datasets generated and/or analyzed during the current study are available from the corresponding author upon reasonable request.
